# Putting interactions on the map

**DOI:** 10.7554/eLife.76801

**Published:** 2022-02-17

**Authors:** César Augusto Ruiz Agudelo, Ángela María Cortes Gómez

**Affiliations:** 1 Programa de Doctorado en Ciencias Ambientales y Sostenibilidad, Universidad de Bogotá Jorge Tadeo Lozano Bogotá Colombia; 2 Facultad de Estudios Ambientales y Rurales, Pontificia Universidad Javeriana Bogotá Colombia

**Keywords:** ecosystem services, nature's contributions to people, multifunctional landscapes, functional connectivity, environmental planning, environmental management, None

## Abstract

A method called functional connectivity mapping helps model some of the complex interactions between multiple ecosystem services.

**Related research article** Field R, Parrott L. 2022. Mapping the functional connectivity of ecosystem services supply across a regional landscape. *eLife* **11**:e69395. doi: 10.7554/eLife.69395

Ecosystem services are defined as “the benefits that humans derive from nature” (MEA, 2005); they link biophysical reality with human well-being. More generally, they can be thought of as the resources and conditions offered by ecosystems that improve human life, ranging from water supplies, food, and materials to recreation, enjoyment of an area, or opportunities to exercise. Human intervention and management can affect the supply of these ecosystem services, for example, by ensuring the supply lasts for a long time, or by consuming it quickly and unsustainably.

Now, imagine a hypothetical landscape with large and small forests, rivers, streams, crop areas, livestock areas, towns, and areas of secondary vegetation (vegetation that grows after disturbances, both human and nature inflicted, such as a flood or a forest being cut down). The land in this area can supply several ecosystem services at different levels simultaneously. To manage this region sustainably, it is important to know how the different ecosystems services interact with each other to determine the best ways to use the land.

Previous work (e.g., Agudelo et al., 2020) recognizes that the reality of ecosystem services is complex, and there may even be a lack of consensus on what constitutes an ecosystem service. However, the connections or interactions between ecosystem services seem clearer, and can be divided into four groups: trade-offs (two ecosystem services show opposing trends), synergy (one ecosystem service increases the benefits of another), bundling or clustering (ecosystem services that appear in regular patterns), and flow (the interaction that describes how supplies flow from the ecosystem to its beneficiaries; Bennett et al., 2009; Hughes et al., 2007; Raudsepp-Hearne et al., 2010; Lee and Lautenbach, 2016).

Modelling and mapping the interactions among multiple ecosystem services should improve the understanding of the benefits that ecosystems can provide to humans. Unfortunately, it is not currently possible to characterize the interactions between ecosystem services in enough detail for decision-makers to make changes to ecosystems with confidence. On the other hand, while some tools have been developed to support decision-making about ecosystem services in specific areas, most of these approaches lack the complexity required to fully answer the questions of when, where, and how nature contributes to ecosystem services (Akçakaya et al., 2016).

Landscape connectivity theory attempts to describe “the degree to which the landscape facilitates movement among resource patches” (Taylor et al., 1993). Now, in eLife, Rachel Field and Lael Parrott, of the University of British Columbia, report an approach to better characterize the interactions between ecosystem services that builds on landscape connectivity theory and existing ecosystem services mapping and modelling (Field and Parrott, 2022). Their methods allow scientists to move away from a static vision of ecosystem services mapping, and measure trade-offs and flow.

The novelty of Field and Parrott’s approach relies on it being replicable in different landscape types with different land-uses by exploiting existing information on the supply of individual ecosystem services. The new method also incorporates landscape connectivity theory, allowing a closer analysis of how resources move between ecosystem services, which cannot be addressed with traditional mapping methods. The characterization of flows specifically, is deepened by going beyond the notion of ecosystem services supply areas, which are static, allowing the identification of corridors through which ecosystem services supplies move, and identifying which ecosystem services depend on each other (Figure 1).

**Figure 1.**
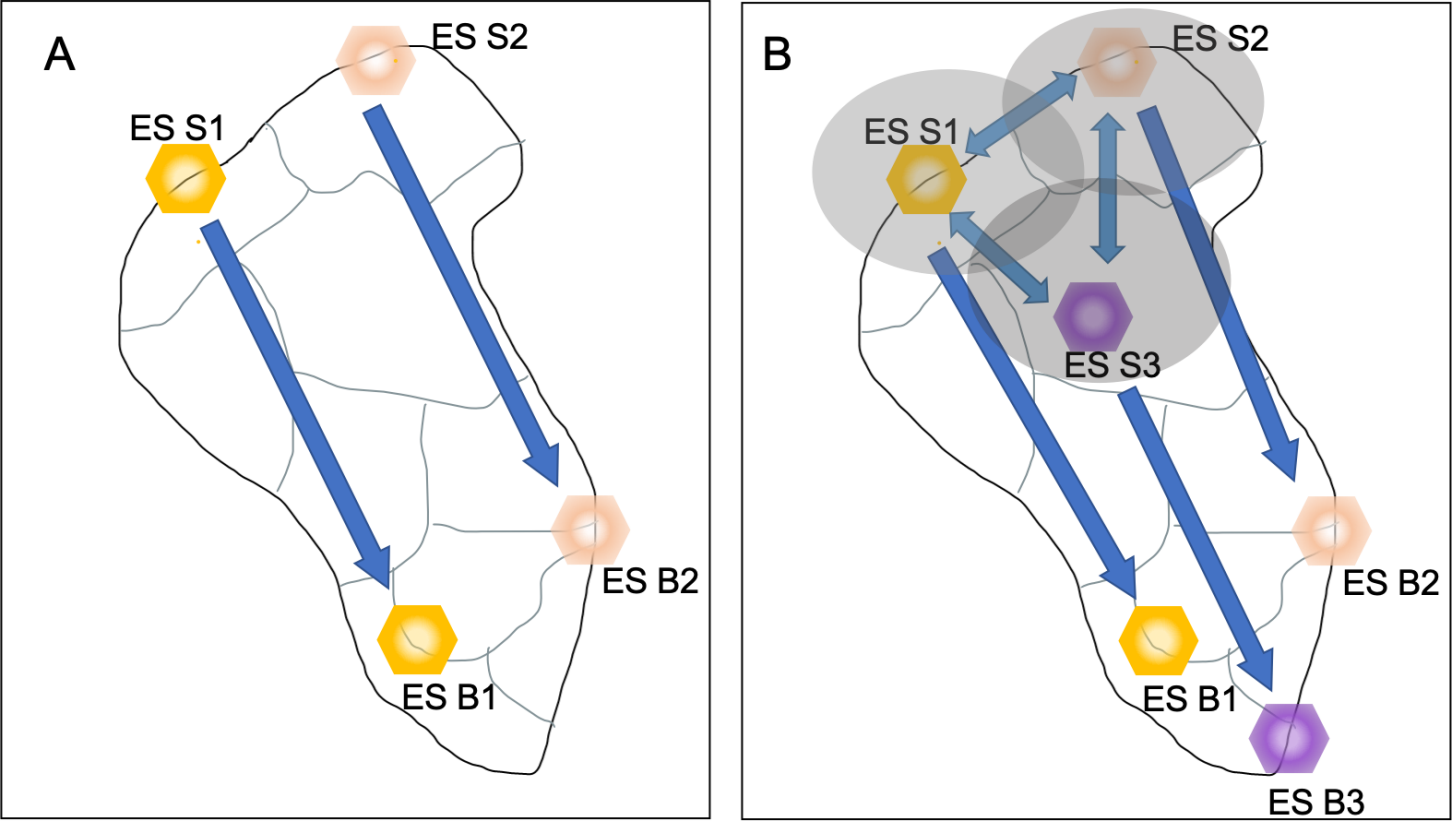
Classical and new approaches to visualizing the interactions between ecosystem services. Different ecosystem services (ES S1, yellow hexagon; ES S2, red hexagon; ES S3, purple hexagon) can produce different social benefits (ES B1, yellow hexagon; ES B2, red hexagon; ES B3, purple hexagon). (**A**) The classical approach to mapping ecosystem services provides a static vision of the landscape. Each ecosystem service is in a fixed position and is only connected to the benefit it produces (arrow with one arrowhead). There are no interactions between the services, and therefore no view of the potential effects that changing how one ecosystem service is exploited could have on other ecosystem services. (**B**) The multifunctional connectivity approach taken by Field and Parrott considers interactions between the different ecosystem services, either in how they physically overlap (yellow, red and purple shaded ellipses) or in how they interact to produce benefits (double headed arrows). This provides a more holistic view of ecosystem services and their benefits.

By providing spatial information on the connectivity between ecosystem services, Field and Parrott’s method enables local and regional environmental planning and management that takes full consideration of the complex and multiscale interactions between ecological processes, land use, land cover, and ecosystem service supply.

Despite these significant advances, future research into mapping ecosystem services still has challenges to face. First, while Field and Parrott incorporate three ecosystem services into their analysis, this is not enough to model real ecosystems, which usually have more than three services. Therefore, it will important to develop methods to incorporate the connectivity of multiple ecosystem services. Second, it will be necessary to overcome the static vision of ecosystem services supply areas, moving on to a more dynamic vision that takes the connections between different ecosystem services into account. Further, Field and Parrott’s approach relies heavily on existing information, but how can their methods be applied to scenarios in which the spatial information about ecosystem services is scarce? Finally, the new methodology allows scientists to measure flows and trade-offs, but it will be important to also measure bundles/clusters and synergies to get a full picture of ecosystem services supply.
